# In Their Own Words: Parents and Key Informants’ Views on Nutrition Education and Family Health Behaviors

**DOI:** 10.3390/ijerph18158155

**Published:** 2021-08-01

**Authors:** Katherine E. McManus, Adrian Bertrand, Anastasia M. Snelling, Elizabeth W. Cotter

**Affiliations:** 1 Department of Psychology, American University, Washington, DC 20016, USA; 2 Department of Health Studies, American University, Washington, DC 20016, USA; adrian.bertrand19@gmail.com (A.B.); stacey@american.edu (A.M.S.); cotter@american.edu (E.W.C.)

**Keywords:** food choices, dietary behaviors, food access

## Abstract

Parents, health professionals, and communities are integral in the development of nutrition behaviors that reduce children’s risk for high body mass index (BMI) and chronic disease. The aim of this study was to conduct formative evaluations with key health informants and parents to understand the specific strategies that families use at mealtimes to promote their family’s health, along with the barriers they face in attending current nutrition education programming. Focus groups (in English and Spanish) were conducted with parents (*n* = 22; 63.64% Black/African American, 13.64% Black but not African American, 18.18% Hispanic/Latinx) whose household was located in a community where 50% of residents’ gross income was ≤185% of the federal poverty level. Semi-structured interviews were conducted with six key informants with expertise in family health and nutrition. Inductive thematic analysis was used to identify themes across interviews. Six general themes emerged from the interviews including perceptions of health, relationships, health behaviors, facilitators, barriers, and desired changes. Across the six themes, participants responded with suggestions for community-based health promotion programs such as incorporating a broader definition of health to better address the individual and systemic barriers that perpetuate health inequities and make healthy eating difficult. Participants identified stress reduction, health literacy, and cooking knowledge as areas of interest for future programming.

## 1. Introduction

Despite the success of many nutrition education programs, barriers and challenges exist that demonstrate the need for further program enhancement, including enhancing participant retention and sustainability of behavior changes over time [1,2]. Barriers associated with healthy eating and living have been well documented for individuals and systems [3,4], yet combating those barriers still presents as a major challenge for many communities and families. These challenges are even more apparent in urban areas where extreme health disparities and inequities exist between wards or districts [5]. Formative evaluation with community health informants and those who engage in nutrition education programs can provide invaluable insight for future programs to better address the gaps that impact short-term and long-term efficacy.

Parents are integral in the development of nutrition behaviors that reduce children’s risk for high body mass index (BMI) and chronic disease [6,7,8,9]. Approximately two thirds of a child’s daily caloric intake come from food stored or prepared in the home [10]. Parent modeling of healthy eating behaviors has been associated with improved diet and decreased risk of high BMI [11,12,13,14]. While most parents understand the need to teach healthy eating habits to their children; almost 50% report difficulty ensuring that their children practice these habits [15]. Parental barriers to implementing healthy eating habits include time constraints, cost, child preferences, feeling undermined by other members of the family, and unhealthy food advertisements [16]. Qualitative research with parents provides deeper insight into the barriers and facilitators of healthy cooking and lifestyle modification at the individual and family levels. Focus groups with mothers described as low income highlighted their perception that it is easier and cheaper to eat unhealthy foods [17,18]. Parents report inability to afford produce despite participation in food assistance programs [18]. Time constraints, including limited time to shop and prepare meals at home, are additional barriers to healthy eating [19,20,21].

Family-centered, hands-on cooking and nutrition programs are widely used strategies to encourage healthy eating habits amongst parents and their children [22]. During these programs, chef instructors and staff teach participants how to read food labels, choose and prepare healthy foods, and support skill building through hands-on activities. Parental involvement, theory-based strategies, and supportive environments (e.g., supplying healthy foods in school) have been associated with more positive behavioral changes in these programs [23,24,25]. Research also supports the use of cultural adaptations that promote engagement and adherence, such as the incorporation of culturally relevant recipes; the use of culturally congruent facilitators, music, and language; the use of a “health at every size” approach to promote body positivity; and recruitment and implementation in community settings [26,27,28].

Although cooking and nutrition programs are widely used, determinants of health such as social, economic, and environmental inequities drastically influence diet and rates of chronic health conditions [29,30,31]. In Washington DC, USA, significant obesity disparities exist, with rates as high as 72% in the most under-resourced wards in the city [32]. Nearly 15% of residents struggle with food insecurity [33] and only 3 out of the 49 full-service grocery stores in DC are located in the areas that report the highest poverty rates [5]. Cooking and nutrition programs’ inability to address these structural and environmental barriers is a shortcoming; however, programs can empower parents to use targeted, controllable strategies that can improve their families’ diet and health despite these challenges.

Formative evaluation can provide in-depth insight into successful strategies and approaches to combat the stressors and structural challenges parents face. Formative research that is grounded in a social ecological approach can also enhance the ability to build community capacity through strategic partnerships and community engagement [28,34]. The involvement of key health informants (e.g., dietitians, health ministers, doctors) can provide valuable insight to better understand the barriers and facilitators of health behaviors from different perspectives across multiple levels. Therefore, gathering qualitative data from community health informants and parents provides varying perspectives necessary to improve family-centered health programming and interventions. The goal of the current formative evaluation was to gather extensive feedback and insight related to health and eating issues from a targeted group of parents and key health informants in Washington DC, USA, to inform and support future sustainable nutrition education programs. These perspectives will allow program professionals and health researchers to better tailor family nutrition and cooking education resources to the unique needs of food insecure residents in Washington DC, USA wards.

## 2. Materials and Methods

We conducted a series of focus group discussions with parents of children and adolescents ages 7–13 years. This age range was selected because school-based nutrition education programs are often offered to children in this age group and parents still have a powerful influence on children’s dietary intake during this period [10]. We also conducted key health informant interviews with community stakeholders in Washington, DC USA, who have specific expertise in family health.

Our team developed a series of interview questions for parents and key health informants. These questions were informed by our team’s previous experience working with Washington, DC USA families; feedback from the funding agency; community input; and previous research [8,9,13,17,18]. Table 1 provides the list of questions.

Participants were recruited across relevant community and school settings in Washington, DC USA. We sent initial emails to schools, medical clinics, non-profits, and affordable housing communities in Washington, DC USA to assess interest in partnering for the project. Interested partners allowed us to place recruitment flyers in their respective locations and recruit on-site during community events. Our community partners included: an affordable housing community, two non-profits, and a medical clinic. Table 2 describes each recruitment site and the participants recruited. Key health informants were recruited via email or phone call with relevant contacts in the field who play a leadership role in improving the health of Washington, DC USA families.

Parents were eligible if: (1) they were age 18 or older; (2) they were the primary caregiver for a child age 7 to 13 years that was eligible to enroll in nutrition education programming (e.g., child is a student in a DC public school); and (3) their child receives free or reduced lunch or their household is located in an eligible census tract (50% of residents’ gross income is <185% of the federal poverty level). Parents were ineligible if they were unable to communicate in English or Spanish or unwilling to be audio-recorded. Key health informants were eligible if they were age 18 years or older, had work experience in a health-related area, and had significant knowledge about the health of families in DC through their community position (e.g., religious leaders; employees of non-profits or medical clinics). Key health informants were ineligible if they were unable to communicate in English or Spanish or unwilling to be audio-recorded.

The research team used SPSS version 23 (IBM Corp., Armonk, NY, USA) to assess frequency and descriptive analyses of participant demographics. Focus groups were audio-recorded and transcribed verbatim. The Spanish-language focus groups were translated into English by a bilingual research team member and double checked for accuracy by another bilingual research team member. Transcriptions were exported into NVivo 12 (QSR International Pty Ltd, Melbourne, Australia) for thematic analysis, the process of identifying patterns of meaning across qualitative datasets [35]. Our approach was inductive, in that the identification of themes was directed by the content of the data rather than preconceived models or concepts. Members of the research team read all of the interview transcripts to become deeply familiar with the content. After this initial review, the research team met to develop an initial set of “codes” for the parent and key informant transcripts. In dyads, members of the research team independently analyzed and coded each transcript and then met with their respective partner to discuss any discrepancies and to reach a final consensus. The team met weekly to review each dyad’s coding and further refine the codebook.

## 3. Results

In total, we held four parent focus groups (*n* = 22). One focus group was conducted in Spanish and three were conducted in English. Table 3 presents the focus group participant demographic data. We also conducted six interviews with key informants. These key informants included a physician, a health minister, a dietitian, two health educators, and a director of school food services. Six general themes emerged from the interviews: Perceptions of health, relationships, health behaviors, facilitators, barriers, and desired changes. Within these themes, various subthemes arose.

### 3.1. Perceptions of Health

Participants discussed their perceptions of health and relevant health issues in their communities. Table 4 describes each theme, subtheme, and provides a representative quote.

#### 3.1.1. Health Definitions

The majority of parents reported that eating habits were central to health (“*What I think is healthy? Water and vegetables and fruit*”). Others described a more holistic definition (“*When you say healthy I think of the whole body not just food…I think of my sleep, my rest, my emotions, all that*”). Similarly, the majority of key informants described a holistic definition of health, as emphasized by this quote, “*Being healthy means…the word ’whole’. W-H-O-L-E. Spiritually, mentally, physically, emotionally, financially, it means everything is balanced and working as it was created to*.” Key informants further discussed the role of the environment and community members’ social networks in shaping health, as noted by the director of school foods, “*It’s a lot more than eating veggies and whole grains. It’s about having a social network, a support system, living in a clean environment, clean air to breathe, having a safe place to live*.”

#### 3.1.2. Health Concerns

Parents primarily cited chronic diseases when discussing health concerns, including obesity, diabetes and heart disease. Several parents also mentioned mental health issues (“*I feel depressed these days.*”). Four of the key informants also briefly discussed health issues that were most prevalent in their respective communities, including diabetes and asthma. The theme of barriers (described below) was commonly intertwined with descriptions of health concerns. As a health educator at a low-cost medical clinic stated, “*We have patients that come...and they are uncontrolled diabetic, and they’re not uncontrolled because they are not taking their medication or checking their blood glucose, it is because they are really stressed out, reasons that are not under their control.*”

### 3.2. Relationships

Participants discussed how relationships with others influenced health and eating behaviors.

#### 3.2.1. Children

Children seemed to have the most profound effect on the health behaviors of parents. Parents explained that meals were heavily influenced by their children’s food preferences and schedules. Most felt it was difficult to ensure that their children were eating healthfully, particularly regarding fruits and vegetables (“*They be irritated eating* [mimicking children] *‘Salad?’ Yeah, they want to put all this chicken and chop meat. No, let’s just eat a regular salad*”). Overall, parents seemed to be more concerned about their children’s health than their own, (“*You don’t think about nutrition until you have children. Now that I have my daughters, it is my third priority*”). Similarly, among key informants, the health educator discussed parents’ central role in children’s nutrition saying, “*Children have no control over what they’re consuming because mom or dad buys the food*.”

#### 3.2.2. Elders

Older adults were also frequently mentioned as influencing health. Specifically, parents’ eating habits were often based on advice from grandparents or aunts. Many of the parents referenced recipes that have been passed down generations. Key informants similarly highlighted the benefits of having multiple generations in a household. (“*I learned that a lot of families in this neighborhood have multiple generations living in the same household…we found out that caregivers like grandma was in the household and cooking every day*”). A general theme was that parents often lack time to prepare healthy, homemade meals, but having an older adult in the home counteracted this.

#### 3.2.3. Medical Professionals

Some of the positive changes that parents implemented came from advice given to them by medical professionals. This typically came as a result of an immediate threat of a chronic condition, such as diabetes (“*I had to change my eating habits. When I went to get a physical, the doctor said so. I have borderline sugar, so I had to*”). However, this did not always correlate with healthy changes (“[a nurse] *advised me to avoid fried foods and meats but I like to eat meats*”).

### 3.3. Health Behaviors

Participants discussed health behaviors in the context of their current health behaviors, changes they had made, and recommendations for future changes.

#### 3.3.1. Current Habits

Parents described a wide variety of current health behaviors, including family mealtime dynamics. While eating habits varied widely between participants, the majority reported that dinner was a home-cooked meal. Recipes typically came from either recipe books, the internet, or other family members. Several reported, “*cooking from the heart*.” Most preferred meals that were easy and quick to cook, especially if their children could get involved and help with the preparation (“*If I had to pick a meal I like to prepare, I think it would be, I don’t eat it, but tacos. They like to help make tacos, and my kids they help me to make…the lettuce and they can do it themselves*”). Others discussed how different schedules and dietary needs meant that everyone ate at separate times (“*It’s like mealtime is chaos. Because you’re coming from work, coming from school, got to do homework, got to get prepared for the next day*”).

#### 3.3.2. Positive Changes

Many parents could recall examples of when they positively changed their health behaviors. Most changes revolved around improving eating habits, *“Eat more vegetables, more greens, less artificial things…eating more naturally*.” Most participants were open to changing their eating habits, whether that meant eating less fried foods or less snacks.

#### 3.3.3. Recommend Changes

Many parents discussed behaviors they believed people should engage in to live healthfully, such as cutting out artificial sugars, drinking water, and eating fruits and vegetables. Mental health came up in the discussion, as well, and participants brought up social support, meditation, and adequate rest as other behaviors that contribute to good health. Four of the key informants similarly discussed recommendations of behaviors related to health that community members could engage in. For example, the health minister stated, “*It can just be little things that you start eliminating and substituting with better stuff*.”

### 3.4. Facilitators

Participants discussed facilitators of health in their household and community.

#### 3.4.1. Household Facilitators

Many of the parents described not purchasing foods and beverages viewed as unhealthy for their homes. A parent commented, “*At home, we don’t buy regular bread. We don’t have churros, we don’t buy soda either.*” The items that they chose to eliminate from their homes varied, from sugary beverages, to chips, to candy. Several caregivers also discussed how they did not fry foods at home, instead choosing to bake, for example. Similarly, the dietitian mentioned community norms around mealtimes, “*When families have the time, they take the time to enjoy food, and take the pleasure of eating together.*”

#### 3.4.2. Community Facilitators

Parents described the traits of local programs that were most helpful, including being “family friendly” and having activity options for children. One program described as particularly effective had staff who were perceived as “*always going the extra mile to help someone*” and a “*positive vibe*” focused on both physical and mental well-being. Parents also described a preference for programs that “*knock on your door*” and are “*persistent on getting you aware of what’s going on*.” The health minister discussed a sense of motivation within their respective community, “*We know we need to do it, we just need to get out there and start making it happen.*” Key informants also discussed the importance of attuning interventions to community members’ cultural norms. A health educator mentioned, “*Nutrition interventions are as efficient as possible, connecting all those dots, culture, language, and the kinds of foods they are eating. I will never take tortillas from my Central American patients*.” Key health informants also highlighted the value placed on interpersonal connections and supportive family relationships in their respective communities (“*The community I work with…really value family and children and friends and partners*”). Finally, key informants described the programming their own respective organizations provided as facilitators to health (“*Tasty Tuesday [is a program] where we taste an ingredient. Like today they’ll be tasting sweet potato chips made from sweet potatoes and then tomorrow they’ll have baked sweet potatoes*”).

### 3.5. Barriers

Parents and key health informants discussed a variety of barriers to healthy eating.

#### 3.5.1. Time

Time was mentioned repeatedly as a barrier for caregivers to participating in nutrition-related programs. (“*These programs are after work. When I get off work, I’m tired, I don’t have time to sit around and listen to a bunch of people who don’t work sit on their butts and complain*”). Some parents also felt that they had no time to prepare healthy food along with all of their other responsibilities (“*I have to get up at 6:45 am and it’s the school routine for my daughter, my routine, and then, later, picking them up…It is like I don’t have time to think*”). Five of the key informants similarly described time as a barrier to healthy eating. As the dietitian noted, “*A lot of times families have a couple of jobs, families aren’t eating together, or getting fast food or from convenience stores*.”

#### 3.5.2. Cost

Parents described high cost as a barrier that prevented them from consistently purchasing healthful foods. As one parent stated, *“So the only foods that we can afford… we’re saying they’re not healthy foods because that’s what we can afford. We can’t afford healthy foods.”* Produce, and especially organic produce, were perceived as expensive and out of reach for some of the participants. Three of the key informants discussed cost as a barrier to healthy eating. One health educator described conversations she had with families visiting a food pantry, “[Parents say]‘*We can afford cans of peas or vegetables, but the kale we can’t afford,’ you know what I mean? So they prefer like the greens and the kale.*”

#### 3.5.3. Community and Societal Barriers

Parents expressed concerns over the foods that their children and grandchildren were being given when out of the home. Cultural norms around food selection were mentioned in the Spanish-speaking group, “*People who are Latino, we’ve never been trained on how to go to the store and look at food to eat. We just see the pretty little bottle, the nice packaging, and we buy it.*” All of the key informants noted that limited access to healthy foods was a barrier to health. As the physician noted, “*There’s not a grocery store for miles…everything’s processed, everything’s canned*.”

#### 3.5.4. Literacy

Four of the key health informants discussed the issue of health literacy as a potential barrier to healthy eating for some community members. The physician described patients’ difficulty in interpreting measurement of foods in recipes, “*Because when you get it to a quarter of this, a third of this, a third quarter of this, half of this, don’t get me wrong, but the numbers can get a bit overwhelming for some of my patients. And I think that’s what deters them from wanting to look into it*.” The dietitian reported preferring the use of infographics and image-based educational material rather than written instructions to improve participants’ understanding.

### 3.6. Desired Changes

Participants discussed personal and community changes they would like to see occur to improve health.

#### 3.6.1. Personal Changes

Many parents saw areas of possible improvement in their own habits, especially when it came to their eating habits. Most acknowledged that, although they knew the general guidelines for healthy eating, they did not always implement these practices (*“I know how to eat right, but I don’t always”*). Parents often referenced the barriers to adopting these practices, such as time and cost, mentioned above. Other participants felt they needed to learn more about what healthy eating entails and how to make healthy choices (“*More recipes. I use the same recipe every day*”).

#### 3.6.2. Community Changes

Parents had a wide range of changes they hoped to see in their communities. Many requested more health and nutrition program offerings, especially on the weekends. A common concern was that programs offered during the week did not accommodate many working individuals’ schedules. Some participants noted that existing programs weren’t advertised effectively to families (“*It’s important for us as a community to communicate with each other and help each other out and we don’t do that enough*”). Similarly, a key health informant discussed the need for programs encouraging family meals, “*Some coaching or some training, whether it’s like a workshop, just how to bond over dinnertime, you know dinnertime can be your time where you explain what happened in your day*.”Another key health informant highlighted the importance of cooking demonstrations and more healthful recipes, but also stated the importance of providing people with “*the food to take home*.” Even further, a key health informant commented on the need to provide more straightforward, adoptable meal preparation strategies in programming (“*Like when we do our recipes for our programs we make sure they’re simple steps, like 5 steps, we wouldn’t have like 15 steps, simple ingredients, no more than 5 or 6 ingredients*”). Key health informants also discussed the need for stress reduction programming and the potential for support groups and regular check-ins, even via text (”*Yes you can do this*,” as suggested by the health minister). Finally, the participants generated creative solutions and recommendations for future programming that addressed inequities in access to food (“*What if we had a mini grocery store in the community? And maybe it’s discounted or something. With fresh produce that you could get at Harris Teeter or Whole Foods or something, but it’s right there in the community*”).

## 4. Discussion

In this research, we examined the health-related strengths, mealtime habits, barriers to healthy eating, and future program interests of a targeted diverse group of Washington, DC USA residents. A strength of this study was our grounding in formative evaluation and stakeholder input. Gaining insight from parents and key health informants who live and work in urban communities where inequities exist between districts allows for deeper understanding of the environmental and community systems and strains that either facilitate or hinder sustainable change. Through this study, parents and key health informants also provided creative solutions, in their own words, to the challenges and stressors they face in everyday life.

While this study supported previous findings regarding facilitators and barriers to healthy living, it also yielded a key emphasis on a broader definition of health relevant to future programming. Throughout our study, our participants explained and emphasized their holistic definitions of health. Our participants stressed the desire for program enhancement such as stress reduction techniques and quick supportive check-ins as facilitators of healthier eating. This holistic definition demonstrated the complexities and interplay between mental well-being and physical health. Previous findings have focused on cost, time, relationships, access, and systematic factors [16,17,18,19,20,21,28,36]; however, our study highlighted the increasing need for stress reduction to help facilitate healthy lifestyle behavior change.

Participants emphasized that stress impeded healthy eating behaviors and parents’ ability to create a healthy environment for their children. Common psychosocial stressors such as financial difficulties, work demands, lack of social support, and balancing work and home can impact one’s ability to modify lifestyle behaviors [36,37]. Berge et al. [38] reported that parents who experience interpersonal conflicts and financial stressors are more likely to engage in unhealthy food-related parenting practices. Our participants described a need for programs that create a positive environment, include stress reduction techniques, and provide an overt focus on fostering supportive connections. Future programs might consider small ways to introduce stress management into their programming or provide resources that families can access for stress management-related resources. Future research could examine the influence of programs that integrate a stress management focus in comparison to more traditional nutrition education programs on parent cooking and feeding behaviors. Even further, future community-based health promotion programs could include broader definitions of health to better address the individual and systemic barriers that perpetuate health disparities and make healthy eating difficult [39,40].

Another important finding to note was the participants desire to increase their health literacy and cooking knowledge. Parents reported that they have basic knowledge about healthy eating, but desire more hands-on skills related to preparing quick and cost-effective meals. Some participants discussed the need to enhance literacy around common recipe ingredients and the interpretation of food measurements. Based on our participants’ feedback, future recipe-related documents might include photos of required ingredients, along with more understandable measurements (e.g., a “handful” of lettuce as opposed to a cup of lettuce). Many parents reported wanting to learn how to prepare a wider range of recipes, along with learning healthy adaptations for their family recipes.

Related to health literacy and cooking knowledge, our participants and key health informants described a preference for videos and visuals rather than text-heavy documents. Key health informants highlighted the use of text messaging in between educational sessions to motivate participants to enact the skills learned. Text messages might reinforce parents’ incorporation of vegetables into recipes or their role modeling of healthy eating behaviors to children. Regarding marketing materials, parents seemed most motivated to participate in programs designed to enhance their children’s health. 

Participants reported a desire to attend programming, if programs were offered in optimal locations. From the parent interviews, it was clear that parents were passionate about their family’s health and receptive to learning new things, yet reported lack of time to attend programs due to work and parenting responsibilities. Our research highlighted the need to meet parents where they are likely to be. Therefore, providing small, meaningful doses of nutrition education in supermarkets, medical clinics, food pantries, farmers markets, and/or affordable housing communities would be more feasible for families with limited time. Programming might include brief 15 min cooking demonstrations or 5 min educational opportunities. 

Participants in our study expressed strongest interest in organizations that wanted to invest in the community and its well-being long-term. They also spoke favorably about programs and organizations that made clear efforts to get to know residents’ personally and provide education and support tailored to their respective situations. Supporting previous research [41], parents in our focus groups praised programs run by organizations that integrated themselves into the community and prioritized the diverse needs of community members. Therefore, future nutrition educational programs should continue to explore strategic partnerships with other likeminded institutions (e.g., local medical centers, non-profits, food pantries, universities) in order to combine resources and create more comprehensive programs, either within or outside school systems.

Some strengths of this study include the wide variety of perspectives drawn from both parents and key informants, as well as the rich detail gathered from participants due to the qualitative design. Furthermore, formative evaluation allowed us to listen first to the target population. A few key limitations of our study should also be noted. First, our study included a small, convenience sample that might not be generalizable to groups across the United States. Future research could replicate our approach across multiple geographic locations, including more rural areas, to strengthen and expand the recommendations from the current study. Another limitation is that our qualitative data is solely from the perspective of parents and key health informants. Future studies might include local policy and community advocacy groups to evaluate potential breakdowns between intentions and execution of programming. Despite these limitations, our findings provide promising recommendations for future programs to consider, including continued focus on experiential activities, expanding program reach beyond school systems, creation of easy-to-understand materials with informational graphics, and curricula that considers stress and mental well-being.

## 5. Conclusions

Based on our results from the parent and key health informant focus groups, future community-based health promotion programs could benefit from including a broader definition of health to better address the individual and systemic barriers that perpetuate health inequities and make healthy eating difficult. Our findings suggest that families desire a holistic definition of health which can be influenced by their familial relationships, access within their communities, and the barriers that impede both individual and systemic levels of change. Parents suggested that small meaningful doses of nutritional education within their communities, holistic programming that considers mental health and well-being, and programs that capitalize on pre-existing partnerships would help improve their health behaviors. Furthermore, there is a desire for programs aimed at improving health literacy and cooking knowledge. Overall, parents are passionate and interested in improving their families’ health; however, programs that are accessible within their communities, encourage holistic definitions of health, address the barriers of cost and time, and teach new cooking skills and nutritional knowledge may be most effective.

## Figures and Tables

**Table 1:** Focus groups and key informant interview questions.

**Parent Focus Group Questions**
What does being healthy mean to you? Where does health fall in your list of priorities? What do mealtimes typically look like in your home? What are strengths that your family brings to mealtimes? Have you changed your eating habits before? ○ If so, what did you change? ○ What factors led to that change? How do you role model healthy behaviors to your children? What is the most helpful health-related resource you have been a part of? Why? ○ How do the activities you (or your child) learned in that resource carry over into your home life? What are your biggest barriers when it comes to participating in health programs? ○ How have you overcome these barriers in the past? What resources would you like to see come to your community related to health? What is your main source of information on healthy eating? When you’re cooking, do you follow recipes? ○ If yes, where do you find recipes? ○ How do you decide how to prepare meals? In what ways do you use technology (e.g., social media, apps, the internet) when you prepare meals? One of our goals is to help families eat healthier. What advice do you have for us? What opportunities or skills related to healthy eating are you most interested in? Of all the things we discussed, what to you is the most important?
**Key Informant Interview Questions**
What does being healthy mean to you? How do you define health in the community you work in? One of our goals is to help families eat healthier. What advice do you have for us? What are meal times for families in your community typically like? What are strengths that families in your community bring to the table when it comes to healthy living? What health-related programs are most needed in the community you work with? What makes members of the community you work in thrive?

**Table 2:** Overview of recruitment sites and participants recruited.

Recruitment Site	Brief Description of Services	Number of Participants Recruited	Number of Focus Groups
1	An affordable housing community located in SE DC	8 parents	1 (conducted in English)
2	A non-profit organization for underserved youth and families located in SE DC	7 parents	1 (conducted in English)
3	A non-profit for underserved women and families located in NW DC	2 parents	1 (conducted in English)
4	A low-cost medical clinic located in NW DC	5 parents	1 (conducted in Spanish)

**Table 3:** Focus group participant demographics.

Demographic Variables	Parents (*n* = 22)
Age (y)	44.18 ± 13.31
Gender (% female)	90.91
Race (%)	
Black/African American	63.64
Black but NOT African American	13.64
Latinx	18.18
Biracial	4.55
Other	0
Education (% parents only)	
Some High School	13.64
High School Diploma	50.00
College Degree	4.55
Graduate Degree	9.09
Unstated	13.64
Number of Children in Home	2.55 ± 1.61

**Table 4:** Key informant and parent focus group themes, subthemes, and representative quotes.

Theme	Subtheme	Definition	Sample Quote
Perceptions of Health	Health Definitions	How health is defined and understood by parents/caregivers and within a community	“Healthy for me is like exercise, eating right. That’s two of the main things.” (parent) “I think that being healthy involves social wellbeing, emotional wellbeing, physical wellbeing, there’s a lot of factors that influence our health. Being healthy is, having access to healthy food, having access to a safe environment at work, having a good paying job to afford housing and your children and your family.” (key informant)
	Health Concerns	Health issues that are concerning to parents and caregivers	“I think soda is a big issue” (parent)
	Health Recommendations	Behaviors of parents/caregivers and key informants believe will improve family health	“Our kids need to know how to eat a lot of vegetables, water.” (parent) “They’re doing what they need to do to go to their doctor’s appointments and the follow up, and they’re taking advantage of the services that are available to them.” (key informant)
Relationships	Children	How children contribute to parent/caregiver health behaviors	“I have a 12-year old, he eats a lot. So we usually do breakfast, snacks, lunch, more snacks, snacks again, dinner, but I try to balance it…I try to introduce new stuff to them.” (parent)
	Elders	How older adults contribute to parents and caregivers’ health behaviors	“Well I usually get the recipes from my daughter’s grandmother on her dad’s side. So she like sends her with a lot of good recipes and clippings or you know she has a book she sends.” (parent)
	Medical Professionals	How medical professionals contribute to parent/caregiver health behaviors	“I went to the doctor and realized I had high cholesterol…and from that time on, I had to start learning how to eat.” (parent)
Health Behaviors	Current Habits	Descriptions of current health habits and norms during mealtimes	“We’ll never really sit at a table together. It’s like we’re separately eating, everybody eating on like their own time. Because I may not even eat until after they already went to bed or something and then I’m gonna fix me a plate.” (parent)
	Positive Changes	Changes parents and caregivers reported making in the past to improve their health	“Changed from fried. We used to fry a lot, so changed from frying to like eating more baked chicken or fish.” (parent)
Barriers	Time	Difficulty preparing healthy meals because of hectic schedules and time constraints	“So when I normally work twelve hours a day, I eat at lunchtime, let’s say, but it’s not like sitting down to eat.” (parent) “Because of the fast way of life and with multiple jobs, moms tend to rely on McDonalds, Burger King, and rely on the food that is given at schools, that involves processed foods, and at lunch, kids are eating pizza…I know parents try to do their best at trying to provide healthy food. I think it has to do with the fast-paced environment.” (key informant)
	Putting Other First	Difficulty prioritizing one’s own health needs over family members’ demands	“That’s why I’ll be sick a lot because I’ll be busy doing stuff for other people.” (parent) “I have women who come to the clinic who want to eat healthy, but she has to prepare a meal for herself and kids and a separate one for her husband.” (key informant)
	Cost	Inability to afford healthy foods	“We can’t afford healthy foods, because if you get organic lettuce, might be $1.50 a pound. Where regular lettuce it’ll be 97 cents a pound, and I got five kids to feed.” (parent) “It has to do with economic status, making enough money to pay rent and put some food on the table. A lot of our patients view eating healthy as more expensive, that is their perception.” (key informant)
	Accessibility	Inability to access services related to health and nutrition	“If I know something is healthy but I don’t have access to it, it is going to be really hard for me to do that.” (key informant)
	Community and Societal Barriers	Aspects of the community and United States’ society that make healthy eating challenging	“The youngest, today he told me this exactly, ‘Mommy, in summer school they give me hamburgers and hotdogs. This isn’t lunch, this is junk food.’” (parent)
	Literacy	Lack of knowledge about healthy eating or inability to easily interpret information about healthy eating	“A lot of our families in our area, they have certain situations you know…Like when we do our recipes for our programs we make sure they’re simple steps, like 5 steps, we wouldn’t have like 15 steps, simple ingredients, no more than 5 or 6 ingredients.” (key informant)
	Safety	Concerns about danger in one’s community that interfere with healthy living	“I think it’s a matter of feeling safe in your community. So that you can get out and walk at any time. That you can walk any time, day or night. Maybe you don’t walk after dark. Or after dark, you want somebody to walk with you. So I think it could be a safety issue, especially for outdoor activities.” (key informant)
Facilitators	Household	Aspects of the home environment that promote health	“I try to get simple snacks like baked chips instead of regular chips, or more fruit.” (parent)
	Community	Aspects of the surrounding community that promote health	“With [name of local clinic], they have nutritionists and I started working with a nutritionist.” (parent) “The community I work with is really resilient and really value family and children and friends and partners” (key informant)
Desired Changes	Personal	Changes parents and caregivers would like to make in their own lives	“I’m actually thinking about vegetables. I don’t eat very many vegetables, but I know that I need to incorporate that into my meals.” (parent)
	Community	Changes parents and caregivers would like to see occur in their surrounding community and future health programming key informants hope to see	“So a lot of times when parents aren’t showing up maybe because they see something on a walk but they don’t understand what it is so we have to use our voices …there are people out there that can’t read and they’re not going to say ‘hey I can’t read.” (parent) “What if we had a mini grocery store in the community? And maybe it’s discounted or something. With fresh produce that you could get at Harris Teeter or Whole Foods or something, but it’s right there in the community” (key informant)

## Data Availability

The data presented in this study are available on request from the corresponding author.

## References

[1] An R., Wang J., Liu J., Shen J., Loehmer E., McCaffrey J. (2019). A systematic review of food pantry-based interventions in the USA. Public Health Nutr..

[2] Robroek S.J., Lindeboom D.E., Burdorf A. (2012). Initial and sustained participation in an internet-delivered long-term worksite health promotion program on physical activity and nutrition. J. Med. Internet Res..

[3] Ronto R., Rathi N., Worsley A., Sanders T., Lonsdale C., Wolfenden L. (2020). Enablers and barriers to implementation of and compliance with school-based healthy food and beverage policies: A systematic literature review and meta-synthesis. Public Health Nutr..

[4] A Brockman T., A Sim L., Biggs B.K., A Bronars C., Meiers S.J., Tolleson E., Ridgeway J.L., Asiedu G.B., Hanza M.M., A Olson M. (2020). Healthy eating in a Boys & Girls Club afterschool programme: Barriers, facilitators and opportunities. Health Educ. J..

[5] DC Hunger Solutions Closing the Grocery Store Gap in the Nation’s Capital. https://www.dchunger.org/wp-content/uploads/2018/11/dchs-closing-grocery-store-gap-report.pdf.

[6] Dallacker M., Hertwig R., Mata J. (2019). Quality matters: A meta-analysis on components of healthy family meals. Health Psychol..

[7] Golan M., Weizman A. (2001). Familial approach to the treatment of childhood obesity: Conceptual model. J. Nutr. Educ..

[8] Lindsay A.C., Sussner K.M., Kim J., Gortmaker S. (2006). The role of parents in preventing childhood obesity. Future Child..

[9] Moore L.C., Harris C.V., Bradlyn A.S. (2012). Exploring the relationship between parental concern and the management of childhood obesity. Matern. Child Health J..

[10] Poti J.M., Popkin B.M. (2011). Trends in energy intake among US children by eating location and food source, 1977–2006. J. Am. Diet. Assoc..

[11] Alia K.A., Wilson D.K., George S.M.S., Schneider E., Kitzman-Ulrich H. (2013). Effects of parenting style and parent-related weight and diet on adolescent weight status. J. Pediatr. Psychol..

[12] Crossman A., Sullivan D.A., Benin M. (2006). The family environment and American adolescents’ risk of obesity as young adults. Soc. Sci. Med..

[13] Natale R.A., Messiah S.E., Asfour L., Uhlhorn S.B., Delamater A., Arheart K.L. (2014). Role modeling as an early childhood obesity prevention strategy: Effect of parents and teachers on preschool children’s healthy lifestyle habits. J. Dev. Behav. Pediatr..

[14] Martinasek M.P., DeBate R.D., Walvoord A.G., Melton S.T., Himmelgreen D., Allen T.D., McDermott R.J. (2010). Using social marketing to understand the family dinner with working mothers. Ecol. Food Nutr..

[15] Robert Wood Johnson Foundation, National Public Radio, Harvard School of Public Health A Poll about Children and Weight: Crunch Time during the American Work and School-Week—3pm to Bed. https://media.npr.org/documents/2013/feb/Children%20and%20Weight_Summary.pdf.

[16] Pechmann C., Catlin J.R., Zheng Y. (2020). Facilitating Adolescent Well-Being: A Review of the Challenges and Opportunities and the Beneficial Roles of Parents, Schools, Neighborhoods, and Policymakers. J. Consum. Psychol..

[17] Dammann K.W., Smith C. (2009). Factors affecting low-income women’s food choices and the perceived impact of dietary intake and socioeconomic status on their health and weight. J. Nutr. Educ. Behav..

[18] Haynes-Maslow L., Auvergne L., Mark B., Ammerman A., Weiner B.J. (2015). Low-income individuals’ perceptions about fruit and vegetable access programs: A qualitative study. J. Nutr. Educ. Behav..

[19] Rawlins E., Baker G., Maynard M., Harding S. (2013). Perceptions of healthy eating and physical activity in an ethnically diverse sample of young children and their parents: The DEAL prevention of obesity study. J. Hum. Nutr. Diet..

[20] Sealy Y.M. (2010). Parents’ food choices: Obesity among minority parents and children. J. Community Health Nurs..

[21] Clayton P., Connelly J., Ellington M., Rojas V., Lorenzo Y., Trak-Fellermeier M.A., Palacios C. (2021). Facilitators and barriers of children’s participation in nutrition, physical activity, and obesity interventions: A systematic review. Curr. Dev. Nutr..

[22] Muzaffar H., Metcalfe J.J., Fiese B. (2018). Narrative review of culinary interventions with children in schools to promote healthy eating: Directions for future research and practice. Curr. Dev. Nutr..

[23] Golley R.K., Hendrie G., Slater A., Corsini N. (2011). Interventions that involve parents to improve children’s weight-related nutrition intake and activity patterns–what nutrition and activity targets and behaviour change techniques are associated with intervention effectiveness?. Obes. Rev..

[24] Lavelle H., Mackay D., Pell J. (2012). Systematic review and meta-analysis of school-based interventions to reduce body mass index. J. Public Health.

[25] Meiklejohn S., Ryan L., Palermo C. (2016). A systematic review of the impact of multi-strategy nutrition education programs on health and nutrition of adolescents. J. Nutr. Educ. Behav..

[26] Barkin S.L., Gesell S.B., Po’e E.K., Escarfuller J., Tempesti T. (2012). Culturally tailored, family-centered, behavioral obesity intervention for Latino-American preschool-aged children. Pediatrics.

[27] Ickes M.J., Sharma M. (2011). Does behavioral intention predict nutrition behaviors related to adolescent obesity?. ICAN Infant Child Adolesc. Nutr..

[28] Kumanyika S., Grier S. (2006). Targeting interventions for ethnic minority and low-income populations. Future Child..

[29] Moerder C., Hamilton L., Alper J., National Academies of Sciences, Engineering, and Medicine Identifying and Addressing Health Inequities in Urban Settings. Health-Focused Public–Private Partnerships in the Urban Context: Proceedings of a Workshop.

[30] Lavizzo-Mourey R.J., Besser R.E., Williams D.R. (2021). Understanding and Mitigating Health Inequities—Past, Current, and Future Directions. N. Engl. J. Med..

[31] Borras A.M., Mohamed F.A. (2020). Health inequities and the shifting paradigms of food security, food insecurity, and food sovereignty. Int. J. Health Serv..

[32] DC Department of Health Obesity. https://dchealth.dc.gov/service/obesity-overview.

[33] DC Hunger Solutions Facts on Hunger in DC. https://www.dchunger.org/hunger-in-dc/.

[34] Bronfenbrenner U. (1981). The Ecology of Human Development.

[35] Braun V., Clarke V. (2006). Using thematic analysis in psychology. Qual. Res. Psychol..

[36] Steptoe A., Feldman P.J., Kunz S., Owen N., Willemsen G., Marmot M. (2002). Stress responsivity and socioeconomic status. A mechanism for increased cardiovascular disease risk?. Eur. Heart J..

[37] Wang J.L. (2006). Perceived work stress, imbalance between work and family/personal lives, and mental disorders. Soc. Psychiatry Psychiatr. Epidemiol..

[38] Berge J.M., Tate A., Trofholz A., Fertig A., Crow S., Neumark-Sztainer D., Miner M. (2018). Examining within-and across-day relationships between transient and chronic stress and parent food-related parenting practices in a racially/ethnically diverse and immigrant population. Int. J. Behav. Nutr. Phys. Act..

[39] Goodman E., Slap G.B., Huang B. (2003). The public health impact of socioeconomic status on adolescent depression and obesity. Am. J. Public Health.

[40] Subica A.M., Grills C.T., Douglas J.A., Villanueva S. (2016). Communities of color creating healthy environments to combat childhood obesity. Am. J. Public Health.

[41] Mayer K. (2009). Childhood obesity prevention: Focusing on the community food environment. Fam. Community Health.

